# Perceived Smile Attractiveness by Undergraduate Students Across Different Disciplines

**DOI:** 10.1155/ijod/6693205

**Published:** 2026-09-22

**Authors:** Kiran Rehman, Kirti Saxena, Sadia Tabassum, Joey Wong, Joslyn Chua Hao Min, Mohammed Nadeem Bijle, Umer Daood

**Affiliations:** ^1^ Division of Restorative Dentistry, School of Dentistry, IMU University, Bukit Jalil, Kuala Lumpur 57000, Malaysia, imu.edu.my; ^2^ Division of Clinical Oral Health Sciences, School of Dentistry, IMU University, Bukit Jalil, Kuala Lumpur 57000, Malaysia, imu.edu.my; ^3^ Department of Paediatric Dentistry, Jinnah Medical and Dental College, Karachi, Pakistan, jmc.edu.pk; ^4^ Department of Orthodontics, Pediatric and Community Dentistry, College of Dental Medicine, University of Sharjah, Sharjah, UAE, sharjah.ac.ae

**Keywords:** attractiveness, dentistry, pharmacy, psychology, smile, students

## Abstract

**Purpose:**

The study aimed to examine differences in perceiving smile attractiveness between undergraduate students across different university disciplines.

**Materials and Methods:**

Undergraduate students pursuing dentistry, pharmacy and psychology volunteered to participate in the study based on an extended invitation by the study contributors. Then, the students were informed about the protocol to follow as participants in the study in a scheduled session, whereby 24 modified images were presented, and smile‐attractiveness ratings were sought on a 6‐point Likert scale. The data were organised to examine differences in smile attractiveness perception among students with respect to factors such as discipline, skin tone in images and the tooth shades presented. The data were analysed using a three‐factor ANOVA with a Bonferroni post hoc test at all levels, with *α* = 0.05.

**Results:**

A total of 106 participants provided their perceived smile attractiveness ratings across the disciplines, dentistry (46), pharmacy (47) and psychology (13), with a mean age of 21.7 ± 1.68 years. A statistically significant difference was observed overall and with independent factors (*p* < 0.05). There was a statistically significant interaction between the disciplines and students’ VITA tooth shade perception, as indicated by smile attractiveness ratings (*p* = 0.002). Dark skin tone was distinctly perceived (*p* < 0.05) by the students (irrespective of discipline); however, the fair and medium skin tones remained undifferentiable in the students’ ratings (*p* > 0.05). The dental students demonstrated the potential to distinctly identify differences in tooth shades, as evidenced by smile attractiveness ratings, compared to students from other disciplines (*p* < 0.05).

**Conclusion:**

Undergraduate students across different university disciplines perceived smile attractiveness differently, with dental students discerning variations between tooth shades.

## 1. Introduction

An attractive personality is significant to several facets of life, including social acceptance, interactions, existence/self‐esteem and cultural inclusivity. Although subjective, facial features provide the primary insight to opine on the attractiveness of an individual [1]. For an aesthetic appeal, several extra‐oral structures must follow a precise ratio, also known as the golden proportion [2]. Further, the appeal is significantly influenced by the smile. Accordingly, several people express concerns about their smile appearance, for which they seek aesthetic dental treatment to improve facial attractiveness, which is perceived as enhancing physical characteristics for social well‐being. Indistinctly, the conception of a smile differs across societies based on their norms, culture, practices, profession, comprehension and outlook, with many other aspects that are not obvious to any ordinary person in their state of being. In the current era, the presence of social media, trending fashion and media influencers/societal role models plays a pivotal role in shaping the notion of how smile attractiveness is perceived [3]. However, to deliver a perceived attractive smile, it is imperative that dentists/aesthetic dentists primarily identify the uniform factors that, admittedly, influence smile attractiveness amongst individuals.

Several factors contribute to smile attractiveness, which undoubtedly include the visual attractiveness of teeth. The attractiveness of teeth is usually associated with their size, form/shape, structure, shade, symmetry, alignment and relation to other teeth [4]. As for laymen, their overall appearance is affected by their dental appearance, principally referring to the shade of their teeth [5]. The tooth colour/shade is comprehended amongst dentists as a vital consideration in prosthesis fabrication, with their training emphasising the need [6, 7]. The tooth shade selection is dependent on several optical factors while largely being influenced by the light absorption/reflection and the viewpoint position, that is, the position from where the teeth are being witnessed [8]. It is irrefutable that other intra‐oral and extra‐oral structures influence the appearance of teeth, including but not limited to gingiva (colour/pigmentation), lips (shape/colour/relation), skin (tone/colour/texture), and physiological changes [9, 10]. Furthermore, drastic changes in tooth shades might be obvious to different people; however, to identify subtle differences, one would need a trained professional eye. Therefore, with several factors affecting tooth shades, whereby the tooth colour is ultimately utilised to improve the smile perception, enhancing attractiveness, it is essential to delineate substantial areas that impart an effect on tooth shades, aiding in efficient aesthetic treatment delivery.

In contemporary times, due to media influence, the perceived smile attractiveness is inclined towards having whiter teeth, especially with the younger generation. With the classical VITA shades (A1‐D4), the lighter shades, being A1/B1, swerve towards whiter/lighter shades, as preferred by the patient pool today [10–12]. Students initially exploring their careers, particularly at an undergraduate level, are naive and subject to active learning. These cohorts are developing concepts and comprehension based on their routine schooling with minimal influences of lateral training. Apart from that, they belong to the younger generation, as referred to above, who prefer whiter teeth. However, their perception of teeth and the differences in shades might not be obviously discernible to students trained in different disciplines. In a study by Wagner et al. [13], laymen differed from the dental technicians and dentists in assessing appearance and tooth shades. Thus, it could be implied that trained comprehension influences the perception of attractiveness applicable to the smile and beyond. This might further be predisposed to the generation factor as the traits observed in different generations are known to be disparate, as outlined with the current media influences. Categorically, it is unknown whether the current generation, with the plethora of information available online, perceives smile attractiveness similarly due to the generation influence or differs, given the training platform in universities.

Previously, a regional study concluded that the natives (predominantly aged 18–30 years) perceived smile attractiveness as being influenced by skin tone and tooth shades, with preferences towards lighter shades. Further, the study did not identify differences in smile attractiveness perception between dentists and non‐dentists [6]. Given that the current generation pursuing university education will be dealing with the global masses in a decade or two, it is imperative to understand their preferences towards a smile being attractive, which could eventually aid clinicians in determining the norm. Additionally, different extra‐ and intra‐oral features can influence smile perception in individuals trained across various disciplines, thereby being pertinent to rule out specific factors that play a role in smile attractiveness, given the training, immediate skin tone and tooth shades in candidates involved with exploring careers in different disciplines. Therefore, the study aimed to examine whether there are differences in perceiving smile attractiveness between undergraduate students across different university disciplines. The null hypothesis tested in the present study was that there is no difference in the smile attractiveness perception of undergraduate students across different disciplines while inferring that the students (irrespective of the disciplines) perceive smile attractiveness similarly when presented with smile images of varying tooth shades and skin tones of a male/female patient.

## 2. Materials and Methods

### 2.1. Study Outline

**Figure 1:**
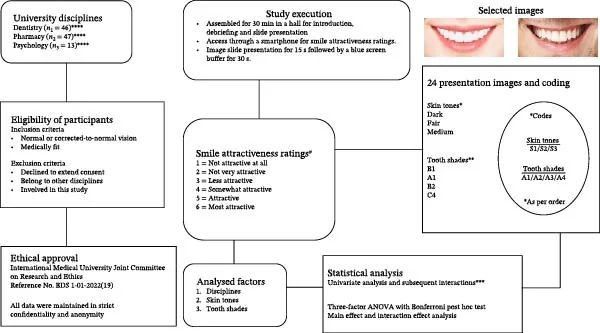
Study outline. The “ ^∗^” denotes skin tones based on Maybelline foundation guide, “ ^∗∗^” denotes tooth shades based on VITA tooth shade guide, “ ^∗∗∗^” denotes Statistical significance determined at *p* < 0.05 for all levels, “ ^∗∗∗∗^” denotes number of participants from Year 3, and “#” denotes 6‐point Likert scale.

The outline of the present study is detailed in Figure 1. Briefly, undergraduate students pursuing dentistry, pharmacy and psychology were extended an invitation to participate in the study. Volunteers participated from these disciplines and were asked to attend a session for the study execution. Then, the students were informed about the protocol to follow as participants in the study, whereby 24 modified images were presented and smile attractiveness ratings were sought on a 6‐point Likert scale. The data were organised to examine differences in smile attractiveness perception between the students with respect to factors like discipline, skin tone in images and the presented tooth shades.

### 2.2. Ethical Review, Approval and Confidentiality

As undergraduate students across different university disciplines were deemed participants in the study, ethical clearance was sought from the International Medical University (IMU) Joint Committee on Research and Ethics. The proposal for the study was reviewed and approved by the committee with clearance as per Reference Number BDS 1‐01‐2022(19) to proceed with the acknowledged protocol. No deviations from the protocol were either encountered or required during the execution of this study. All data were maintained in strict confidentiality in regard to the anonymity of the participants, with all received responses.

### 2.3. Study Participants

An invitation was extended to the undergraduate students of the university in the disciplines of dentistry, pharmacy and psychology to participate in the study. It was explained to students that the participation was entirely voluntary, and their consent was needed before they could be enrolled in the study. For standardisation, Year 3 students from their respective programmes were deemed eligible to participate in the study. The following criteria were followed:

#### 2.3.1. Inclusion Criteria

a. Students with normal or corrected‐to‐normal vision.
b. Students who declared themselves as medically fit with no acute/chronic condition leading to difficulty in their well‐being that will affect their participation.

#### 2.3.2. Exclusion Criteria

a. Students who declined to provide consent to participate in the study.
b. Students from disciplines other than dentistry, pharmacy and psychology.
c. Students involved in the study assistance.

### 2.4. Smile, Skin Tones and Tooth Shades

**Figure 2:**
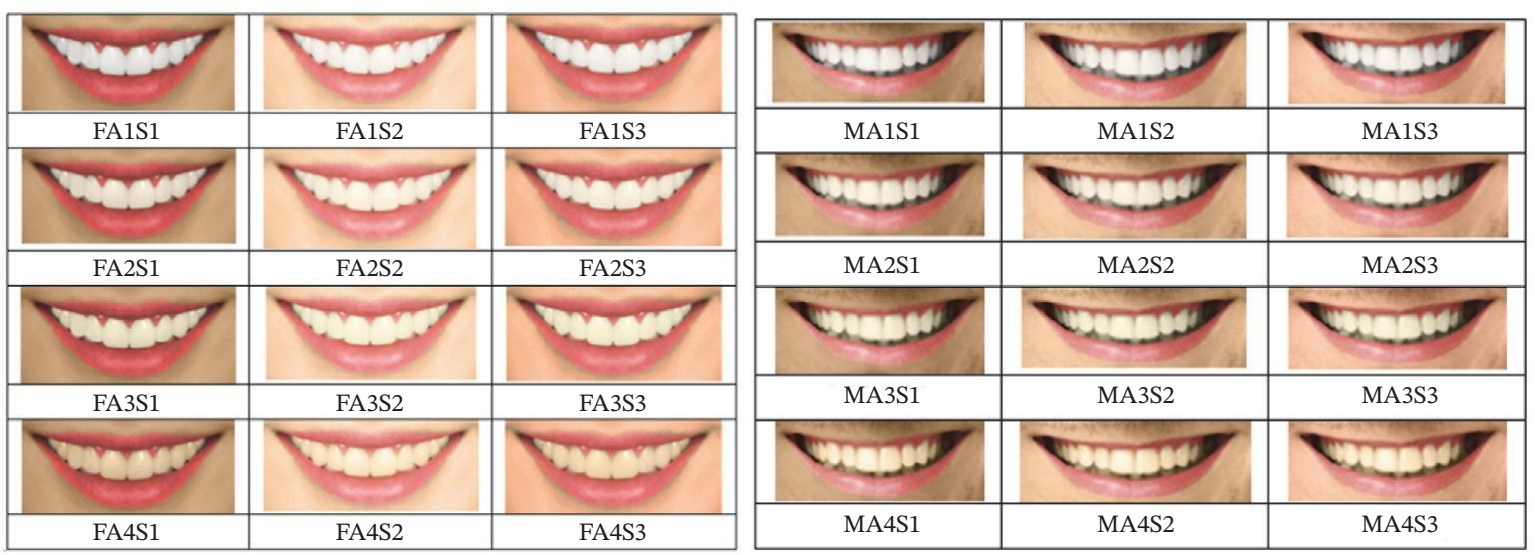
Photos presented to students with different skin tones and tooth shades. M/F—represents smile in a M—male and F—female volunteer. A1−4 are VITA tooth shades (VTS) representing as A1—VTS B1; A2—VTS A1; A3—VTS B2; A4—VTS C4. S1−3 are skin tone changes as S1—dark; S2—fair; S3—medium.

Volunteers were sought to consent to their smile images across the university. Eventually, two images (one male and one female) captured under standardised conditions were identified that showed no clinically significant malocclusion or deviations from the perceived ideal smile by the investigators, who were all dental professionals. Images were then modified using Adobe Photoshop CS5 Extended (v. 12.0 x64, Adobe Systems Inc., California, USA) to represent changes in the following: (1) skin tone based on the Maybelline foundation guide (i.e., fair/medium/dark) and (2) tooth shades according to the VITA tooth shade guide (A1, B1, B2, C4). A total of 24 images were arranged for the study participants to rate their perception of smile attractiveness under uniform experimental conditions (Figure 2).

### 2.5. Image Presentation

The images were assigned unique identification codes based on the identified two images, skin tones and the above‐listed VITA tooth shades. The details of the codes are presented in the Figure 2 legend. The details on the codes remained unrevealed to the participants throughout the study. The images were then included in PowerPoint (Microsoft, Redmond, USA) slides, with a blue screen slide serving the purpose of a visual buffer between images. The image display duration was auto‐maintained for 15 s, and the blue screen duration was set at 30 s.

All participants were asked to assemble in a large hall. The active participation time by the students was 30 min, which included introduction, debriefing and slide presentation for smile attractiveness ratings. The students were then asked to access their smartphones with the shared link to complete a generated Google Form as they moved along the presentation slides. The Google Form had initially listed questions, viz., the student discipline and the age of the student in years, for which the students were given time until they all completed these initial sections. Then, the students were subjected to the presentation slides to complete their ratings and eventually submit the responses. The students were not given a second chance to review the images.

### 2.6. Smile Attractiveness Ratings

The smile attractiveness ratings were sought using a 6‐point Likert scale, as listed below:

1 = Not attractive at all
2 = Not very attractive
3 = Less attractive
4 = Somewhat attractive
5 = Attractive
6 = Most attractive

As we moved higher on the numbers in the scale, it emphasised that the smile was attractive, while being the converse at lower orders. The Likert scale was listed alongside the image identification codes in the Google Forms, allowing students to input their ratings.

### 2.7. Statistical Analysis

The responses received from the participants were entered in an MS Excel spreadsheet (Microsoft, Redmond, USA), which was further exported to SPSS v. 30 (SPSS Inc., IBM, Chicago, USA) for statistical analysis.

For analysis, the ratings were treated as scale variables to subject the data to univariate factor analysis and subsequent interactions across three independent variables: Factor 1, disciplines; Factor 2, skin tones; and Factor 3, VITA tooth shades.

Three‐factor ANOVA with the Bonferroni post hoc test at all levels was employed to determine the effect of independent factors and their interactions on the smile attractiveness ratings by the undergraduate student participants in the study.

The significance was determined at *p* < 0.05 for all analytical levels.

## 3. Results

A total of 106 participants responded with their perceived smile attractiveness ratings across the disciplines: dentistry (*n*_1_ = 46), pharmacy (*n*_2_ = 47) and psychology (*n*_3_ = 13). The mean age of the participants was 21.7 ± 1.68 years. All the participants submitted their responses for all displayed images, with no areas rendered blank during data analysis.

### 3.1. Perceived Smile Attractiveness by Students

The analysed main and interaction effects (as *F*‐statistics) of all factors, that is, disciplines, skin tones and VITA tooth shades, on the smile attractiveness ratings are shown in Table 1. A statistically significant difference was observed overall, and with independent factors (*p* < 0.05). There was a statistically significant interaction between the disciplines and the VITA tooth shade perception of the students, as outlined by smile attractiveness ratings (*p* = 0.002). Other interactions at 2‐factors/all factors were statistically not significant for any further analysis (*p* > 0.05), implying further exploration of the main factor effects and significant interaction effects.

**Table 1:** Univariate analysis on smile attractiveness ratings by the students.

Factors and interactions	*F*‐statistics	*p*‐Value^a^
Overall	19.19	<0.001
Factor 1: disciplines	6.91	0.001
Factor 2: skin tones	46.85	<0.001
Factor 3: VITA tooth shades	88.20	<0.001
Disciplines × VITA tooth shades	3.44	0.002
Disciplines × skin tones	1.99	0.093
Toothshades × skin tones	0.82	0.553
All factor interactions	1.00	0.447

^a^Three‐factor ANOVA with Bonferroni post hoc test; *p* < 0.05 is significant.

### 3.2. Effect of Factors on the Smile Attractiveness Ratings by the Students

The main factor effect analysis of all independent factors on the smile attractiveness ratings by the students is shown in Table 2.

**Table 2:** Factor effect analysis on smile attractiveness ratings by students.

Factors	Mean estimates	Std. error	*p*‐Value^1^
Disciplines			
Dentistry	3.65^a^	0.03	0.001
Pharmacy	3.82^b^	0.03	
Psychology	3.66^ab^	0.06	
Skin tones			
Dark	3.34^a^	0.05	<0.001
Fair	3.86^b^	0.05	
Medium	3.93^b^	0.05	
VITA tooth shades			
B1	4.02^a^	0.05	<0.001
A1	4.12^a^	0.05	
B2	3.71^b^	0.05	
C4	3.00^c^	0.05	

*Note*: Different superscript lowercase English alphabets (a, b, c) demonstrate significant differences between groups under each independent factors.

^1^Three‐factor ANOVA with Bonferroni post hoc test; *p* < 0.05 is significant.

For disciplines, the mean estimate with perceived smile attractiveness for pharmacy students was significantly higher than dental students (*p* < 0.05). However, no significant difference could be observed in the ratings of psychology students and the students of other disciplines (*p* > 0.05), suggestive of overlaps in perceived smile attractiveness.

Further, the dark skin tone was distinctly perceived (*p* < 0.05) by the students (irrespective of disciplines); however, the fair and medium skin tones remained undifferentiable with the students’ ratings (*p* > 0.05). Additionally, the fair/medium tones were considered more attractive by the students than the dark skin tone (*p* < 0.05)

With the main effects analysis on VITA tooth shades, overall, the students perceived A1 and B1 shades as similar (*p* > 0.05), while B2 and C2 were distinguishable from each other and A1/B1 shades (*p* < 0.05).

### 3.3. Smile Attractiveness Ratings by Students From Different Disciplines on Tooth Shades

The interaction effect analysis between disciplines and tooth shades based on the students’ ratings is presented in Table 3. The students from the dental school could differentiate between tooth shades, with the most attractive being A1 and lesser attractive amongst the tooth shades reported as C4 (*p* < 0.05). While the pharmacy students perceived A1 and B1 as similar shades, given the higher smile attractiveness ratings (*p* > 0.05) and identified C4 and B2 as distinct from all independent shades (*p* < 0.05). The students from the psychology discipline perceived smile attractiveness with B1, A1 and A2 shades as similar (*p* > 0.05), which was significantly higher than the C4 shades, suggesting that B1/A1/A2 shades are more attractive while C4 shades are less attractive.

**Table 3:** Interaction effect analysis between disciplines and tooth shades based on smile attractiveness ratings by the students.

Disciplines	VITA tooth shade	Mean estimates	Std. error
Dentistry	B1	3.94^a^	0.07
	A1	4.29^b^	0.07
	B2	3.60^c^	0.07
	C4	2.78^d^	0.07

Pharmacy	B1	4.22^a^	0.07
	A1	4.15^a^	0.07
	B2	3.83^b^	0.07
	C4	3.10^c^	0.07

Psychology	B1	3.91^a^	0.13
	A1	3.92^a^	0.13
	B2	3.69^a^	0.13
	C4	3.12^b^	0.13

*Note*: Different superscript lowercase English alphabets (a, b, c, d) demonstrate significant differences between groups (VITA tooth shade) under each discipline.

^∗^Three‐factor ANOVA with Bonferroni post hoc test; *p* < 0.05 is significant.

Collectively, the results of the present study suggest that there is a difference in perceiving smile attractiveness between undergraduate dental students across different university disciplines (*p* < 0.05), with the dental students demonstrating the potential to distinctly identify differences between different tooth shades as comprehended using the smile attractiveness ratings (*p* < 0.05).

## 4. Discussion

In this study, we examined whether there are differences in perceiving smile attractiveness between undergraduate students across different university disciplines by recruiting participants through an extended invitation. The factors analysed to estimate the effect on smile attractiveness were disciplines, skin tones and tooth shades, with two identified images which were perceived as an ideal smile by the study investigators [10, 14, 15]. The results of the study explain that the smile attractiveness was perceived distinctly by students of different disciplines, with dental students efficiently discerning the differences in tooth shades. Further, the students perceived lighter tooth shades as more attractive, given the generation that comprehends whiter smiles as attractive. In addition, the results of the study also delineate that training has an impact on how an individual perceives a smile, whereby subtle differences can be identified exclusively by a professionally trained eye. Given the results of the present study, the null hypothesis of the study was rejected and the alternative hypothesis was accepted, confirming that smile attractiveness was perceived differently by undergraduate students across different university disciplines.

The study results can aid dentists/clinicians in extending their smile design, operative care, or prosthetic services to the cohort‐like in the present study. Apart from that, the study explains the need to understand the profession of their patients when delivering dental care. The findings from the present study also confirm that active learning or training influences the perceived smile attractiveness [16]. A dental student might be able to differentiate between the shades and would prefer shades as appropriate to the skin tone and personality [6, 7]; however, students trained in disciplines other than dentistry would possibly understand lighter shades as more attractive and vice versa with the darker shades in the VITA tooth shade guide, thereby unwilling to accept dental work in such shades, although being even with the previous dental records [14]. While these are suggestive elements and limited to the results of the present study, the clinicians must always look at the societal, cultural and personalised preferences of their patients as not all patients are uniform in their perception, conception and comprehension with respect to their regional domains [17].

As an initial observation on the distribution of participants across different disciplines, it is explicit that the number of participants from the psychology discipline was lower than the other disciplines. The psychology school students at the university were fewer in number, and hence, there was an obvious difference. While looking at the participants in the study and the number of students in their respective disciplines at Year 3, it appears that the proportion of participants was still maintained at 33%, credibly justifying the representative samples from the student pool for this study. However, it is advisable to include a larger participant count to improve the generalisability of the study results. Furthermore, a statistically significant difference with Factor 1: Disciplines highlights the differences between the disciplines, whereby the ratings from the students in the psychology school did not significantly differ from the other schools, explaining that the participant count had a limited effect on discerning differences in the ratings between students of different disciplines.

Referring to the factor analysis, it is well recognised that overall, the students were able to identify differences between the skin tones based on their smile attractiveness ratings; however, the differences were limited to differentiating between dark and fair/medium, whereby no differences between fair and medium skin tones were observed. While looking at the images, it can be appreciated that the medium skin tone is a tone lower than fair on the tone spectrum and a pronounced difference is perceptible when fair and dark tones are compared with ordinary vision. Therefore, the smile attractiveness ratings were lower for the dark skin tones, as they are more deeply pigmented than the fair/medium skin tones. With fair and medium skin tones as per the Maybelline foundation guide, the fair skin tone is described as the lightest skin tone with a very light and often pale appearance, whereas the medium skin tone is considered not so pale being regarded as neutral. Hence, a stark difference remains unnoticeable as the tone is on a continuous spectrum with limited changes in pigmentation, often regarded as subtle. It is also notable that skin tone preference is subject to the respective population and differs with regions, beliefs, culture, and most importantly, individual preferences and perceptions [18–20].

Given the study outcome, it is evident that the dental students perceived smile attractiveness distinctly with different tooth shades, with lighter shades deemed attractive than the darker shades. However, the students from pharmacy and psychology demonstrated overlaps with shade differentiation, as determined by their attractiveness ratings. The pharmacy students perceived shades A1 and B1 both as the most attractive, and the psychology students perceived A1, B1 and B2 as attractive. For A1/B1, it could be due to the shades being the lightest in their shade band. Here, it also implies that it is the perception of similar chroma to their respective hues that endures as the confounding factor for students in professions other than dentistry. For psychology students, it is argued that an additional B shade belonging to the same hue might have compromised the perception, further relating to the minimum number of participants from the discipline. However, these explanations are confined to the basic understanding of hue, chroma and value within the tooth shades. Exploring further, the pharmacy students understand human biological aspects better than the psychology students, whereby the focus is on behavioural and social influences upon people in psychology. Therefore, the apparent differences between the pharmacy and psychology students are due to their training as pharmacy students indulge in a deeper understanding of human biology, with teeth being a biological element.

All factor interactions and interactions between disciplines–skin tone and tooth shade–skin tone were statistically not significant in the study analysis. Although it is known that skin tone affects the choice of colour, however, the philosophy is inapplicable to tooth shades and the overall smile attractiveness when multiple factors are being assessed [21]. Hereby, it is derived that disciplines, skin tones and tooth shades as cumulative entities demonstrate a limited effect on smile attractiveness, whereby the primary factors discern an effect on how an individual perceives a smile, whereas the factor interaction is limited to the disciplines and tooth shades explored in the present study. Furthermore, smile attractiveness is markedly influenced by teeth and therefore, when factors like skin tones and disciplines are exclusively upheld for estimating smile attractiveness, a true effect remains unidentified. This is in agreement with the results of a previous study, whereby a brighter tooth shade significantly influenced the perception of a smile, independent of skin tone [12]. The rationale for the skin tone and tooth shade, with no significant interaction, is infused in a similar perception of fair and medium skin tones by the undergraduate students, which could also be concurrent with the maturity of the students. Accordingly, any further exploration of the study area must focus on individuals with diverse populations, whereby confounders like age groups, maturity, regional diversity, societal perception, to name a few, are controlled to comprehend factors that affect smile attractiveness [16, 22].

The present study aligns well with the study objectives; however, the generalisability of the study results still requires input from a larger and more diverse population, as this was a single‐centre study and limited to a few disciplines. Substantially lower participation from students pursuing psychology than from those in dentistry and pharmacy would have affected statistical power as sample size is an integral component of sample‐power analysis. Furthermore, participation was restricted to Year 3 students, while factors such as variations in maturity, exposure and educational experience might yield distinct insights from other undergraduate and postgraduate students, which remain unidentified. Apart from the factors studied, other factors might play a role in the perception of smile attractiveness, which were not analysed in this study, including but not limited to the immediate peri‐oral structures like the gingiva and lips, which were seen as factors affecting skin tone in previous studies [20, 23–25]. Additionally, the inclusion of dynamic interactions was not considered as a factor determining real‐time experience that affects an individual’s perception. Last, since the study was cross‐sectional, it implies perception at a single point in time.

While here, the study results explicitly highlight that an interaction effect between skin tone, tooth shades and disciplines does not exist, it explains that when determining smile attractiveness, the focus of an individual is on teeth and specifically on tooth shades. However, suppose the immediate peri‐oral structures are included in examining the smile attractiveness, and there is a notable influence; then, this might further provide insights to decide whether one needs lip cosmetic treatments or aesthetic gingival surgeries along with restorative/prosthetic care for smile enhancement, eventually defining attractiveness. Also, with future studies, it is recommended to investigate areas listed above and whether counselling or providing information on the subject to individuals trained in disciplines other than dentistry changes their perception of smile attractiveness, as then the counselling routine could be implemented with patients seeking dental care for aesthetics.

## 5. Conclusions

Based on the results of the present study, it can be concluded that

a. Undergraduate students pursuing studies across different university disciplines perceived smile attractiveness differently.
b. The dental undergraduate students discerned variations in tooth shades than students in other disciplines.

## Author Contributions

**Kiran Rehman**: conceptualisation, methodology, writing – reviewing and editing. **Kirti Saxena, Sadia Tabassum, Joey Wong and Joslyn Chua Hao Min**: investigation, data curation, writing – reviewing and editing. **Mohammed Nadeem Bijle**: data analysis and interpretation, writing – original draft preparation. **Umer Daood**: conceptualisation, supervision, writing – reviewing and editing.

## Funding

The research was supported by the IMU Joint Committee on Research and Ethics (Grant BDS I‐01‐2022(19)).

## Ethics Statement

This study was reviewed and approved by the IMU Joint Committee on Research and Ethics (Ethics Committee/IRB Reference Number BDS I‐01‐2022(19)). Consent was sought from the participants prior to commencement of the study.

## Conflicts of Interest

The authors declare no conflicts of interest.

## Data Availability

The datasets used and/or analysed during the current study are available from the corresponding author upon reasonable request.
